# Complex mechanisms linking neurocognitive dysfunction to insulin resistance and other metabolic dysfunction

**DOI:** 10.12688/f1000research.8300.2

**Published:** 2016-06-02

**Authors:** Luke E. Stoeckel, Zoe Arvanitakis, Sam Gandy, Dana Small, C. Ronald Kahn, Alvaro Pascual-Leone, Aaron Pawlyk, Robert Sherwin, Philip Smith

**Affiliations:** 1 National Institute of Diabetes and Digestive and Kidney Diseases, National Institutes of Health, Bethesda, MD, USA; 2 Rush Alzheimer’s Disease Center, Rush University Medical Center, Chicago, IL, USA; 3 Icahn School of Medicine and James J. Peters VAMC, New York, NY, USA; 4 Yale University School of Medicine, New Haven, CT, USA; 5 Joslin Diabetes Center, Harvard Medical School, Boston, MA, USA; 6 Berenson-Allen Center for Noninvasive Brain Stimulation and Division for Cognitive Neurology, Beth Israel Deaconess Medical Center, Harvard Medical School, Boston, MA, USA

**Keywords:** Diabetes, insulin resistance, obesity, cognition, cognitive impairment, Alzheimer’s disease, vascular dementia, mechanism

## Abstract

Scientific evidence has established several links between metabolic and neurocognitive dysfunction, and epidemiologic evidence has revealed an increased risk of Alzheimer’s disease and vascular dementia in patients with diabetes. In July 2015, the National Institute of Diabetes, Digestive, and Kidney Diseases gathered experts from multiple clinical and scientific disciplines, in a workshop entitled “The Intersection of Metabolic and Neurocognitive Dysfunction”, to clarify the state-of-the-science on the mechanisms linking metabolic dysfunction, and insulin resistance and diabetes in particular, to neurocognitive impairment and dementia. This perspective is intended to serve as a summary of the opinions expressed at this meeting, which focused on identifying gaps and opportunities to advance research in this emerging area with important public health relevance.

## Background

Emerging data have established links between systemic metabolic dysfunction, such as diabetes, and neurocognitive impairment, including dementia. The current epidemic of dementia is driven, at least in part, by the concurrent epidemics of obesity, insulin resistance, diabetes, and metabolic syndrome. Early research sought to elucidate the cause(s) for the apparent role of metabolic dysfunction in the increased prevalence of neurocognitive dysfunction and dementia, tentatively attributed to vascular contributions to cognitive impairment and dementia (VCID), and Alzheimer’s disease (AD).

More recent research has revealed that the relationships linking metabolism and brain dysfunction are bidirectional. On the one hand, diabetes increases risk of dementia by about two-fold, while on the other hand, the development of neurocognitive disorders has been linked to an increased risk of metabolic disease ( Biessels *et al.*, 2014). While the relationships linking metabolism and brain dysfunction in humans are more complex and more difficult to study in isolation, mouse models of isolated cerebral amyloidosis and cerebral amyloid angiopathy have been discovered to have peripheral insulin resistance ( Ruiz *et al.*, 2016).

Genetic, epigenetic, environmental, and other mechanisms (likely in combination) are being implicated as underpinning these relationships and, in some cases, with behavior acting as a disease modifier. Of particular interest, both peripheral insulin resistance (the central pathophysiologic feature of type 2 diabetes) and “brain insulin resistance” (a much-discussed but poorly characterized entity) have been suggested to play important roles in neurocognitive dysfunction and dementia. Insulin resistance and the associated metabolic dysregulation can be driven by high-fat (“Western”) diet, physical inactivity, and obesity, and have deleterious effects on neurocognition ( Ishii & Iadecola, 2015a).

Because the pathophysiological processes that lead to metabolic and neurocognitive dysfunction precede the development of clinical syndromes by years, early intervention and preventive measures are possible and an area of intense study ( Norton *et al.*, 2014). Indeed, AD neuropathology is detectable more than 10 years before the onset of the first clinical symptoms ( Bateman *et al.*, 2012) and pre-diabetes also precedes diabetes by years in most cases. There is now a focus on biomarkers and intervention research at pre-disease states, as there may be a higher chance that diseases are modifiable at this stage and prevention may be attainable ( Sperling *et al.*, 2015).

Confounding the picture is evidence that diabetes worsens VCID and, in turn, that VCID increase the likelihood of developing AD pathology. Nonetheless, diabetes and dementia are complex diseases, with heterogeneous risk factors, underlying mechanisms, and clinical expressions, often with different contributing co-morbidities.

In this era of precision medicine, it is imperative that we identify the various metabolic-neurocognitive phenotypes in order to understand the mechanisms that drive these diseases so that we can develop targeted therapeutic strategies to successfully manage and, hopefully, prevent these complex, multifactorial diseases.

In July 2015, the National Institute of Diabetes and Digestive and Kidney Diseases (NIDDK) at the National Institutes of Health (NIH) gathered a group of cross-disciplinary scientific experts in Bethesda, MD for purposes of:

(1) Reviewing the state-of-the-science;
(2) Identifying key knowledge gaps and critical unanswered questions; and
(3) Developing ideas for future directions to advance investigation at the intersection of metabolic and neurocognitive dysfunction.

The meeting focused on clarifying leading candidate mechanisms that may explain relationships between metabolic and neurocognitive dysfunction, with a special emphasis on the bidirectional relationships between diabetes and/or insulin resistance (either peripheral, central, or both) and cognitive impairment and AD and/or VCID. Although there has been increased attention to the neurodegenerative and/or vascular contributions to cognitive impairment and dementia ( Snyder *et al.*, 2015), it is important to consider the contributions of mechanisms that may not fall into either category as well as mechanisms that span multiple categories.

In this White Paper, we have attempted to survey and summarize the opinions among the experts in attendance with regard to the most likely mechanisms to investigate in order to improve our understanding of diseases and disorders at the interface of metabolism and cognition; in other words, to address the questions of “what do we know now?” and “what directions should be pursued in order to advance that knowledge?”. In so doing, we aim (a) to improve public health at the level of community medical care that considers patients within the context of their behaviors and environment; and (b) to inform public policy that would be required for a major effort toward prevention of these common co-morbid conditions of high societal burden.

## Summary of current knowledge

### Diabetes and neurocognitive dysfunction (including dementia)

The high prevalence of type 2 diabetes (~26%) and dementia (~11%) in individuals > 65 years old, and the increased relative risk of all-cause dementia (~2 to ~2.5-fold) conferred by type 2 diabetes make understanding this connection a major public health imperative. Even individuals with type 2 diabetes without dementia have subtle impairment in performance (a third of a standard deviation, compared to individuals without diabetes) in a range of cognitive functions, including learning and memory, processing speed, and executive function across the lifespan ( Biessels *et al.*, 2014). These decrements in cognitive function are accompanied by structural and functional brain changes, with one estimate attributing five years of brain aging (based on volumetric magnetic resonance imaging, MRI) to diabetes relative to chronological age and estimates in those without diabetes ( Franke *et al.*, 2013). The most common neuroimaging findings include modest white and gray matter atrophy (most pronounced in the temporal and frontal cortices), cerebral small vessel disease, and disrupted structural and functional connectivity ( Biessels & Reagan, 2015). Of relevance to understanding pathophysiologic mechanisms linking diabetes and neurocognition is that some of these brain changes may be observed prior to the development of diabetes ( Brundel *et al.*, 2014; Convit *et al.*, 2003).

The neurocognitive profile of diabetes shares features with aspects of cerebrovascular disease and AD. However, it is still unclear if the relationship between diabetes and neurocognitive dysfunction and dementia is due to classic AD pathology, vascular processes such as infarcts or vessel pathology, other pathologies less closely related to AD or vascular disease (e.g., impaired brain insulin signaling), or some combination of these. Some have proposed that “all-cause” dementia be employed in order to avoid the implication that we can classify etiologies accurately based on current knowledge. Understanding the mechanism(s) that explain the neurocognitive complications of diabetes and other metabolic disease will be important if we are going to be successful in developing therapeutic targets and approaches to mitigate the effects of metabolic disease on the brain and cognitive function. This is especially the case if metabolic disease impacts the brain in ways that differ from its effects on other end organs impacted by diabetes and other metabolic disease.

The CNS pathology associated with metabolic disease has largely been characterized as a cognitive disorder with most of the research focused on brain regions and networks that support higher-order cognitive functions. Research on the central regulation of energy balance and peripheral metabolism, on the other hand, has almost exclusively focused on the hypothalamus and brainstem, brain regions and networks whose contributions to higher-order cognitive functions may not have been fully defined. The recent appreciation of the bidirectional relationship between diabetes and neurocognitive dysfunction/dementia presents an opportunity to combine efforts and resources to tackle both of these complex and related problems together. Cerebral amyloidosis can precede clinical cognitive decline for up to 30 years, and during this early phase may occur in limited regions of the brain. Therefore, it is plausible that AD pathology (amyloid-beta or Aβ, tauopathy) may be present in the hypothalamus prior to the onset of cognitive symptoms, and that this hypothalamic pathology could disrupt homeostatic functions such as energy balance and peripheral metabolism prior to the onset of detectable cognitive dysfunction ( Ishii & Iadecola, 2015b).

### Mechanisms linking diabetes to dementia

While diabetes is known to increase risk for dementia, the underlying mechanisms linking these conditions is less clear. Undoubtedly, given the well-established micro- and macro-vascular complications of diabetes, the least disputed mechanism of brain injury involves cerebrovascular disease. Indeed, it is now well-established that diabetes, and elevation in the most frequently used biomarker of diabetes (hemoglobin A1c), increases the risk of stroke, and that stroke in turn, increases the risk of cognitive impairment and dementia, including vascular dementia ( Abbott *et al.*, 1987; Banerjee *et al.*, 2012; Gorelick *et al.*, 2011; Ivan *et al.*, 2004; Li *et al.*, 2012; Ramirez *et al.*, 2015). Aside from brain infarction, the central pathologic feature of stroke ( Arvanitakis *et al.*, 2006a), other vascular and related processes in the brain plausibly play a pathophysiologic role in relating diabetes to dementia, including white matter disease, breakdown of the blood brain barrier, inflammation, and others ( Hsu *et al.*, 2012; Shimizu *et al.*, 2013). Also, co-morbid conditions to diabetes, such as hypertension, hypercholesterolemia, and obesity, and complications of diabetes such as myocardial infarction, can lead to cerebrovascular disease and dementia, either alone or in concert with one another ( Yaffe, 2007). The published literature as a whole suggests about a two-fold increased risk of stroke in individuals with diabetes, pointing to other pathways also being involved in linking diabetes to dementia.

While AD pathology is often cited to be present in about two-thirds of patients with dementia, this estimate includes both patients with AD pathology alone, as well as those with both AD pathology along with other pathologies such as the pathology of VCID and/or other neurodegenerative dementias (e.g., synucleinopathy, TDP-43, etc). Overall, the most common form of dementia is “mixed dementia”, and that mixture is most commonly one of both vascular and neurodegenerative pathology ( Schneider *et al.*, 2007). Links between diabetes and AD pathology are also reported but remain poorly understood. Some data from *in vivo* biomarker studies such as those using neuroimaging and cerebrospinal fluid, suggest that persons with diabetes have more brain atrophy, reduced glucose metabolism, and other changes in markers of neurodegeneration, including alterations in cerebrospinal fluid and phosphorylated tau in particular, that are in keeping with AD ( Baker *et al.*, 2011; Moran *et al.*, 2015; Roberts *et al.*, 2014). Mice with knockout of the insulin receptor in the brain show increased tau phosphorylation ( Schubert *et al.*, 2004). Data from postmortem brain tissue from elderly individuals have demonstrated the presence of some biochemical features reminiscent of insulin resistance in the hippocampal formation of persons with AD (with and without diabetes) compared to cognitively normal controls, and this insulin resistance has been reported to be associated with pathology that involves both Aβ oligomers and fibrillar amyloid plaques ( Talbot *et al.*, 2012). Consistent with this is the report that a mouse model that accumulates only Aβ oligomers showed metabolic flexibility that remained relatively intact while another mouse model that accumulated both Aβ oligomers and fibrils developed features of insulin resistance ( Ruiz *et al.*, 2016). A more recent postmortem study has shown that “brain insulin resistance” (a term in need of a precise definition) is associated with tau pathology in AD as well as other neurodegenerative tauopathies such as corticobasal degeneration ( Yarchoan *et al.*, 2014). Finally, some genetic data now suggest a link between diabetes and AD, as in the case of the insulin degrading enzyme gene or, more recently the SorCS1 gene which is thought to regulate Aβ metabolism ( Lane *et al.*, 2010; Lane *et al.*, 2013). Thus, amyloidosis, tauopathy, typical AD pathology and/or other neurodegenerative mechanisms -- as well as “brain insulin resistance” (perhaps even apart from peripheral diabetes *per se*) -- appear to play roles in linking diabetes to dementia, apart from the cerebrovascular mechanisms.

Nonetheless, neuropathological studies have largely failed to confirm a consistent association between diabetes and AD pathology. Indeed, only a few studies suggest associations with increased AD pathology, including in subsets of persons such as those who are apolipoprotein E epsilon 4 carriers ( Malek-Ahmadi *et al.*, 2013; Matsuzaki *et al.*, 2010; Peila *et al.*, 2002), but most studies show either no relationship or a relationship with decreased pathology that is modulated by antidiabetic therapies ( Ahtiluoto *et al.*, 2010; Alafuzoff *et al.*, 2009; Arvanitakis *et al.*, 2006a; Beeri *et al.*, 2005; Heitner & Dickson, 1997; Janson *et al.*, 2004; Nelson *et al.*, 2009; Sonnen *et al.*, 2009). There are reports that the clinical cognitive syndrome in diabetes is more dysexecutive rather than amnestic ( Arvanitakis *et al.*, 2004; Arvanitakis *et al.*, 2006b; Nandipati *et al.*, 2012), arguing against a strong diabetes-AD connection. In the largest clinicopathological study to date of about 2,400 persons, diabetes was not associated with AD pathology when assessed using overall measures of AD and more specific measures of severity of tangle and amyloid pathology ( Abner *et al.*, 2016). Whether some individual features of AD pathology (e.g., Aβ oligomer and/or fibril accumulation) play roles remains unclear. In summary, while an AD mechanism is less established (or perhaps less clinically important) than the vascular mechanism, further elucidation of this pathway may bring insight into other opportunities for treatment and prevention of neurocognitive dysfunction and dementia. Along these lines, both observational studies and experimental clinical trials have examined the role of conventional and unconventional (e.g., intranasal) administration of anti-diabetes medications for treatment of clinical and pathologic AD ( Beeri *et al.*, 2008; Craft *et al.*, 2012; Gold *et al.*, 2010; Watson *et al.*, 2005).

Chronic hyperglycemia has been associated with reductions in endothelial glucose transporters such as GLUT1 that could lead to glucose deprivation and excitotoxicity, and could account for some of the neurocognitive complications of diabetes ( Gjedde & Crone, 1981; Matthaei *et al.*, 1986). This is unlike diabetic retinopathy, which has been associated with upregulation of GLUT1 glucose transporter ( Kumagai *et al.*, 1996) and is another example of how diabetic complications in the brain may differ from other organ systems.

Yet other mechanisms underlying the association of diabetes with neurocognitive dysfunction and dementia need to be further explored. While not a widely accepted concept, some researchers suggest that some AD may even be considered a metabolic disease caused by insulin resistance in the brain, separate from diabetes ( Rivera *et al.*, 2005), raising yet again the need for an evidence-based definition for “brain insulin resistance”. Given the range of effects of insulin in the brain, including neurogenesis, neurite outgrowth, and modulation of catecholamine release/uptake, other possible links between insulin resistance and dementia need to be considered. For examples, mice lacking insulin signaling in brain have been shown to have altered dopamine turnover associated with behavioral changes ( Kleinridders *et al.*, 2015). Downstream effects from insulin resistance, whether peripheral or central, could involve increased glucose and advanced glycation end products (AGEs), and alterations in adipokines (e.g., leptin) and other factors ( Cherbuin *et al.*, 2012; Holden *et al.*, 2009; Yaffe *et al.*, 2011). Inflammation-related events in the periphery and brain may be another important factor explaining the connection between diabetes and neurocognitive dysfunction (see ( De Felice & Lourenco, 2015; Ferreira *et al.*, 2014) for recent reviews of this topic). Effects of insulin and other factors, including inflammation in the brain, need further consideration as plausible underlying mechanisms linking insulin resistance, with and without diabetes/pre-diabetes, to dementia.

Even in the absence of vascular and other complications, diabetes can alter synaptic plasticity in the mouse hippocampus resulting in cognitive deficits ( Stranahan *et al.*, 2008), and mice with diabetes are less likely to recover from stroke due to impaired neuroplastic mechanisms ( Sweetnam *et al.*, 2012). Ongoing studies in humans support the hypothesis that cognitive dysfunction in type 2 diabetes is related to alterations in the mechanisms of cortical brain plasticity (Fried *et al.*, unpublished observations). The mechanisms for altered synaptic plasticity in diabetes are unclear but may be linked to reductions in efficacy of N-methyl-D-aspartate receptor (NMDAR)-dependent mechanisms of plasticity. Any downregulation in post-synaptic NMDARs to moderate this risk would consequently reduce the efficiency of long-term potentiation and alter any NMDAR-dependent measures. Alterations in the efficacy of the mechanisms of plasticity have also been identified as early pathophysiologic steps in Alzheimer’s disease and these may thus represent another link between metabolic disorders and dementia.

### Pathophysiology unique to diabetes and its impact on neurocognition

Studies of the relationships linking diabetes, insulin resistance, and neurocognitive dysfunction have largely focused on how peripheral metabolism impacts brain function. Unlike other complications of diabetes (e.g., neuropathy, retinopathy, nephropathy), neurocognitive complications of diabetes have not clearly been demonstrated to correlate with measures of peripheral glycemia (with the exception of very poor glycemic control, HbA1c > 10% and a potential role for glycemic variability) and there is only limited evidence for a modest association with other measures of peripheral glucose regulation (e.g., insulin concentration, insulin action, insulin resistance) ( Geijselaers *et al.*, 2015). It is possible that lower levels of hyperglycemia (i.e., HbA1c < 10%) may have detectable effects on neurocognitive function in adequately powered studies or that HbA1c levels may not reflect highly fluctuating blood glucose concentrations, which could have independent deleterious effects on glucose-sensitive tissues such as brain endothelium. It is also possible that there are central mechanisms that will better account for the neurocognitive dysfunction observed in diabetes and other metabolic disease. One promising line of research has central insulin and insulin-like growth factor 1 (IGF-1) in the spotlight. There is evidence that disrupted central insulin and IGF-1 signaling may lead to disrupted neurotransmitter (e.g., dopamine) and astroglial cell function, brain endothelial cell function involved in formation and regulation of blood-brain barrier (BBB), mitochondrial metabolism and oxidative stress, regulation of the phosphorylation of microtubule-associated tau protein and clearance of Aβ and/or amyloid fibrils, cholesterol synthesis in the brain (important for myelination and membrane function), glucose and lipid metabolism in select regions of the brain, and regulation of central energy balance, which could relate to both metabolic and neurocognitive dysfunction ( Bingham *et al.*, 2002; Brüning *et al.*, 2000; Convit *et al.*, 2003; Kleinridders *et al.*, 2015; Liu *et al.*, 2013; Montagne *et al.*, 2015; Schubert *et al.*, 2004; Stouffer *et al.*, 2015; Suzuki *et al.*, 2010; Suzuki *et al.*, 2013). Restoring insulin function in the brain via intranasal insulin is now being tested as a potential therapeutic option for neurocognitive dysfunction in both diabetes and dementia separately ( Claxton *et al.*, 2015; Novak *et al.*, 2014) and repurposing diabetes drugs for use in dementia has also been proposed ( Yarchoan & Arnold, 2014). However, more research in this area is critically needed. For example, it has been suggested that the reduced insulin signaling observed in aging, and exacerbated in AD, results from a neuroprotective mechanism ( Steculorum *et al.*, 2014). This view derives from evidence that decreased insulin signaling promotes life-extension in *C. elegans* ( Kenyon *et al.*, 1993) and that genetically induced insulin resistance has positive effects on cognition in an animal model of AD ( Killick *et al.*, 2009). If so, then intranasal insulin treatment may produce short term benefits but deleterious effects in the long term. The apparent paradox between pathological insulin resistance and the benefits of acute disruption of insulin signaling is a complex topic requiring further research attention. 

The physiological neuroendocrine mechanism(s) that may participate in the intersection between diet, obesity, diabetes, and dementia is less clear; however, there are a few intriguing mechanisms to consider. Insulin modulates synaptic plasticity of dopamine neurons to regulate reward function and food intake behavior ( Stouffer *et al.*, 2015), as well as behaviors of anxiety and depression ( Kleinridders *et al.*, 2015) and hyperinsulinemia may lead to ineffective regulation of dopamine and increased food intake ( Liu *et al.*, 2013). It is also possible that hormonal changes that occur in obesity and diabetes, such as disrupted glucocorticoid function, could impact synaptic plasticity and brain function ( Bocarsly *et al.*, 2015). Impaired neuroplasticity itself, via a glucocorticoid-related or other mechanism(s), could account for metabolic and neurocognitive dysfunction ( Oberman & Pascual-Leone, 2013; Pascual-Leone *et al.*, 2011; Stranahan *et al.*, 2008; Wosiski-Kuhn *et al.*, 2014). There is also evidence that corticotropin-releasing factor regulates cerebral amyloid accumulation along an as yet undefined pathway that is glucocorticoid-independent ( Macauley *et al.*, 2015).

Microglial dysfunction has become a central point for investigation in AD, since about one-third of genes associated with AD are expressed at high levels or exclusively by microglia. Glucocorticoids appear to impact microglia activation and the release of pro-inflammatory cytokines, which may result in neuroinflammation and cognitive dysfunction ( Dey *et al.*, 2014; Erion *et al.*, 2014; Hao *et al.*, 2016). Somewhat unexpectedly, the gut microbiome has been recently revealed as a potential modulator of physiological function of microglia, and research in other neurodegenerative diseases and Parkinson Disease in particular, have suggested a gut-brain mechanism of disease ( Erny *et al.*, 2015).

### Mechanisms linking diet, obesity, and neurocognition

There has been even less appreciation for how diet and obesity may adversely impact brain function in the absence of diabetes or dementia. Obesity has been associated with altered brain structure and function in animal models and in metabolically and neurologically healthy adults and children ( Bocarsly *et al.*, 2015; Hsu & Kanoski, 2014; Yau *et al.*, 2014). Compared to adults with normal body mass index (BMI = 18.5 – 24.9), overweight and obese adults in midlife (ages 40–59 years) have a relative risk (RR) of 1.35 (overweight) and 2.04 (obese) for AD and 1.26 (overweight) and 1.64 (obese) of any cause dementia ( Anstey *et al.*, 2011). This association between overweight and obesity is not observed in late life (ages 60 years or older) at a time when weight loss is more common and a risk factor for dementia ( Anstey *et al.*, 2011). Consumption of a high fat diet (HFD) can also negatively impact the brain and cognitive function well before obesity onset ( Hsu & Kanoski, 2014). Indeed, obesity can impact cognition independently from metabolic disease and diet can impact metabolic function and cognition independently of obesity. Recent data suggest that weight reduction may even improve neurocognitive function ( Horie *et al.*, 2016). It will therefore be important to look beyond disease states to examine the influence of diet and obesity on neurocognitive function more generally.

Although obesity is occasionally associated with global measures of brain atrophy ( Brooks *et al.*, 2013; Enzinger *et al.*, 2005; Raji *et al.*, 2010) and cognitive decline, many studies suggest that executive function and learning and memory are most affected in both adults and children ( Fitzpatrick *et al.*, 2013; Francis & Stevenson, 2013; Gunstad *et al.*, 2007; Sabia *et al.*, 2009). Corresponding with this neuropsychological profile, structural changes are observed in the parietal and prefrontal cortex (important for executive function), as well as the entorhinal cortex and hippocampus (important for learning/memory) ( Enzinger *et al.*, 2005; Fotuhi *et al.*, 2012; Hao *et al.*, 2016; Miller & Spencer, 2014; Pannacciulli *et al.*, 2006; Raji *et al.*, 2010). These changes may be a consequence rather than a cause of obesity, as supported by neuroimaging data in the minipig animal model ( Val-Laillet *et al.*, 2011). Cerebral blood flow, a marker of neuronal activity, is significantly lower in the dorsolateral and anterior prefrontal cortex in minipigs with diet induced obesity compared to their lean counterparts. Likewise, in humans, there is reduced cortical thickness in the prefrontal, temporal and parietal cortex in adults but not in children ( Sharkey *et al.*, 2015), suggesting that structural changes occur after chronic HFD, metabolic disease and/or obesity. Teasing apart these three factors is an important avenue for future work.

HFD and obesity are also associated with dopamine-dependent mesocorticolimbic-prefrontal alterations that may impact reward learning, motivation, and executive functions ( Johnson & Kenny, 2010; Sevgi *et al.*, 2015; Stice *et al.*, 2008; Sun *et al.*, 2015; Vainik *et al.*, 2013). Overweight/obese compared to healthy weight individuals show reduced change in striatal D2R binding potential in response to glucose ingestion (consistent with reduced dopamine release) and several studies have reported a negative association between basal metabolic index and the blood oxygen level dependent (BOLD) response to milkshake consumption in the dorsal striatum. Although BOLD does not directly measure dopamine release, the effect is dependent upon the Taq1a A1 polymorphism, which affects D2 receptor density, thus linking the BOLD response to abnormal D2R signaling which can also be linked to heightened impulsivity ( Babbs *et al.*, 2013). Along this line, there are emerging data that suggest specific macronutrients or combinations of macronutrients have selective effects on dopamine adaptations. One can speculate on the development of “vicious cycles” that lead to obesity, driven by both genetic makeup, dietary composition, and other factors.

Similar to the association between diabetes and AD, “brain insulin resistance” may play a key role. Insulin action directly affects central glucose metabolism and is posited to influence learning and memory by modulating synaptic plasticity, density, neurotransmission and adult neurogenesis. As such, intranasal administration improves cognition in healthy individuals ( Benedict *et al.*, 2004), raising the possibility that the reverse is also true; that is, decreased insulin signaling associated with sub-clinical insulin resistance accounts for the cognitive dysfunction observed following a HFD or obesity in otherwise healthy individuals. Supporting this hypothesis, a functional type of “brain insulin resistance” has been associated with hypo-active responses in obese non-diabetic, cognitively intact, people within fronto-parietal circuits, which are critical for working memory (the ability to hold and manipulate new and old information “on-line”). Working memory is markedly impaired in overweight and obese individuals and insulin sensitivity mediates a negative association between BMI and response in the fronto-parietal circuit during a working memory task ( Gonzales *et al.*, 2010). Diet-induced obesity can also influence insulin resistance in the hippocampus and likely elsewhere in the brain where insulin receptors are expressed, such as the amygdala and midbrain ( McNay *et al.*, 2010). Thus, metabolic alterations may impact brain function well before the onset of metabolic disorders, and weight reduction may improve both metabolic state and cognition ( Horie *et al.*, 2016).

Typical peripheral insulin resistance is often correlated with a number of other factors associated with diet-induced obesity that may also cause neurocognitive dysfunction. Obesity and HFD lead to systemic and central inflammation with elevated circulating IL-12 and IL-6, both of which have been linked to impaired processing speed and executive function, even independently of metabolic risk factors ( Gregor & Hotamisligil, 2011; Thaler & Schwartz, 2010; Trollor *et al.*, 2012). Thus, several groups have highlighted inflammation as “the pathway to cognitive impairment” ( Miller & Spencer, 2014; Steculorum *et al.*, 2014). Hypersecretion of glucocorticoids may also contribute. The hypothalamic-pituitary-adrenal axis, which is altered in obesity, regulates glucocorticoid secretion, and has in turn been associated with mood changes, memory impairment, and reduced hippocampal volume ( MacQueen & Frodl, 2011; Raber, 1998).

Emerging work also suggests that diet-induced BBB disruption may provide a mechanistic link involving diet, obesity and hippocampal-dependent cognitive functions ( Hsu & Kanoski, 2014). Rats fed a HFD for 90 days show decreased expression of BBB tight junction proteins and the presence of sodium fluorescin, a fluorescent-tagged molecule normally excluded from the brain by the BBB and choroid plexus, was found in the hippocampus but not in the prefrontal cortex or striatum ( Kanoski *et al.*, 2010). More recently, Davidson and colleagues found sodium fluorescin in the hippocampus of rats that failed to show a reversal of diet-induced impairments in hippocampal-dependent tasks upon HFD discontinuation, while rats demonstrating recovery of function did not ( Davidson *et al.*, 2012). Some evidence indicates that BBB integrity may influence learning and memory by affecting the passage of nutrients and neuroendocrine signals such as insulin, leptin and ghrelin from the periphery to the brain.

### Neurocognitive dysfunction affecting food intake behavior and obesity

Many of the brain adaptations produced by diet, obesity and metabolic dysfunction lead to neurocognitive and behavioral changes that may confer additional risk for obesity ( Higgs *et al.*, 2012; Higgs, 2015a; Higgs, 2015b; Sellbom & Gunstad, 2012; Vainik *et al.*, 2013), leading to what has been described as a “vicious cycle of obesity, metabolic disease, and cognitive decline” ( Davidson *et al.*, 2014). Insensitivity to satiety can disrupt habituation or the drop in appetitive responses to foods that accompany eating and there is evidence that the hippocampus plays a role ( Small *et al.*, 2001). Work in rodents has also focused on the role of the hippocampus in sensing the internal state. In an associative learning model, diet-induced damage to the hippocampus may disrupt the ability of interoceptive satiety states to serve as contextual stimuli signaling that a food is not rewarding in a sated state ( Davidson *et al.*, 2014). This, in turn, leads to positive energy balance by promoting eating in the absence of hunger.

The decreased responses in fronto-parietal circuits and corresponding executive function impairments have long been implicated in inhibitory control of feeding behavior ( Alonso-Alonso & Pascual-Leone, 2007; Le *et al.*, 2006). Adaptations in these circuits may therefore further promote overeating, especially in the modern food environment where self-control is especially taxed. In addition, decisions to eat depend upon multiple interacting factors besides hunger and hedonics. Habits, time of day, heath concerns, peer pressure, and stress are amongst the other important modulators. The prefrontal cortex is critical for integrating these factors so that long-term goals can override hedonic and homeostatic signals.

Thus, while strong evidence links obesity to brain dysfunction, the extent to which this arises directly from adiposity, diet, metabolic dysfunction, or other factors is incompletely understood and future research in this area is needed.

## Some research areas in need of increased attention

### Early development

The role of early development, in particular, appears to be critical in understanding the link between obesity, metabolic disease, and neurocognitive dysfunction. Maternal obesity and metabolic disease, and diet during pregnancy may impact the child *in utero* and lead to a greater risk for childhood obesity, metabolic dysfunction, and neurodevelopmental disruption ( Linder *et al.*, 2015; Rivera *et al.*, 2015). High-fat feeding of pregnant and nursing mice has been shown to have important effects of hypothalamic neurocircuit formation that impacts adult brain function ( Vogt *et al.*, 2014). Intervention in early development is critical as there is now evidence that dysregulated metabolic function and obesity lead to detectable brain and cognitive changes in young children, may disrupt academic performance, and could lead to challenges in transitioning to independence and successful adulthood ( Mauras *et al.*, 2015; Mazaika *et al.*, 2016; Semenkovich *et al.*, 2015; Yau *et al.*, 2012; Yau *et al.*, 2014).

### Type 1 diabetes

While the association between type 2 diabetes and dementia has been reported for some time, there has been less attention paid to whether type 1 diabetes may be associated with dementia. We now know that childhood-onset type 1 diabetes leads to detectable brain and cognitive changes in early life through middle adulthood ( Mauras *et al.*, 2015; Mazaika *et al.*, 2016; Nunley *et al.*, 2015a; Nunley *et al.*, 2015b; Ryan *et al.*, 2015; Semenkovich *et al.*, 2015), including changes in brain fMRI ( Musen *et al.*, 2008). It will be important to understand how glycemic dysregulation over the lifecourse impacts neurocognitive function, disease management, and risk for dementia. Iatrogenic hypoglycemia may play some role(s) that remain to be clarified.

### Relationships among central and peripheral insulin signaling and metabolism

A complex and confusing literature has already accumulated wherein no obvious single, simple model adequately explains the bidirectional relationships linking central and peripheral insulin signaling and metabolism. Because of this, we are not attempting to summarize all that literature here. However, we would refer the reader to Ishii & Iadecola (2015b), ( Ruiz *et al.*, 2016), and ( Knight *et al.*, 2016). The former provides an outstanding summary of the primary and secondary involvement of the hypothalamus in AD, while the latter two provide examples of the largely unappreciated metabolic dysfunction present in standard models of AD pathology and the unexpected effects that can result when either the AD pathology, the metabolic pathology, or both, are manipulated.

## Broad research needs to advance the field

The workshop concluded with discussion of the research presented and reviewed above in order to identify research needs to move the field forward. There were several research needs identified at the workshop; however, there were three major themes that emerged:

1. **An urgent need for prioritizing best candidate mechanisms and defining constructs:** Despite a relatively high incidence of obesity, diabetes, and dementia and extensive research into the underlying mechanisms of these diseases independently from one another, there is still a dearth of research at the intersection of these diseases. Advancing research in this area is hampered by the lack of consensus on which of the candidate mechanisms that explain the intersection of obesity, diabetes, and dementia, are most promising to pursue for future research studies. There is also a lack of consensus on how best to define and measure these diseases and/or the aspects of these diseases that may explain their comorbidity. For example, there is a need for an operational definition for “brain insulin resistance” in order to inform decisions about which tools can be leveraged or developed for future research studies to elucidate the potential contribution of “brain insulin resistance” to cognitive impairment and peripheral metabolic dysfunction.
2. **Experimental studies:** There is a crucial role for tissue-based and non-human animals studies to help us elucidate the pathogenesis and treatment of obesity, diabetes, and dementia, including the capability for in depth measurement of central and peripheral processes of critical importance to obesity, diabetes, and dementia at temporal and spatial scales not possible in human studies; however, these studies also have limitations, such as questionable translational potential to human studies. It is a challenge to the researchers that will be advancing this field to address the limitations of their models by developing better models and alternative methods to improve generalizability for the human studies informed by these experimental studies.
3. **Human studies and research collaborations:** It will be important to develop a common vocabulary for defining and categorizing obesity, diabetes, and dementia, and the critical mechanisms that underlie the links between these diseases. It will also be crucial for researchers to interact and collaborate across disciplines from fields such as basic and systems biology, endocrinology, neuroscience and behavioral science, and computational and engineering sciences in order to advance research in this inherently multidisciplinary area requiring the use of multidimensional research approaches and tools. Future research will require more sensitive and specific instruments as well as tests to assess the important contributing factors that explain the neurocognitive complications associated with obesity and diabetes (especially in the initial stages of these diseases) in order to inform the development of treatment protocols that will prevent or mitigate these complications. It will also be critical to evaluate a range of cognitive and brain outcomes using a diverse set of tools and measures, which may be differentially impacted by the various factors associated with neurocognitive dysfunction in the context of metabolic disease (e.g., high-fat diet, visceral adiposity, hyperglycemia, insulin resistance). A holistic vision would allow the research community to identify a range of comorbid and confounding factors associated with obesity and diabetes from vascular risk factors and inflammation to important psychiatric conditions, such as depression. Finally, it is unclear which interventions will lead to the prevention and/or mitigation of the neurocognitive complications associated with obesity and diabetes whether it be repurposed diabetes drugs; diet, weight loss, and exercise interventions; or novel therapeutic approaches such as neuromodulatory or other therapies. This is another research avenue in need of increased attention.

For additional identified broad research needs identified at the workshop, see Supplementary File 2: Other Broad Research Needs.

## Future directions

**Figure 1.**
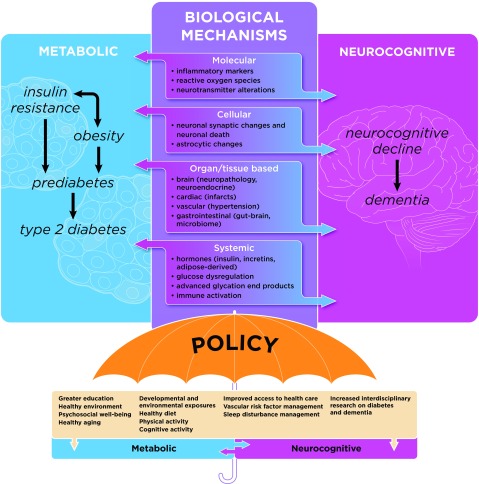
Possible mechanisms explaining the bidirectional relationship between metabolic and neurocognitive dysfunction. The most plausible biological mechanisms (middle panel: molecular, cellular, organ and tissue-based, systemic) are shown that may explain the bidirectional relationship between metabolic (left panel: obesity, insulin resistance, prediabetes, type 2 diabetes) and neurocognitive dysfunction, including dementia (right panel: e.g., VCID, VaD, AD, other). Genetic and epigenetic mechanisms (not represented in the figure) likely trigger the pathophysiological processes underlying metabolic and neurocognitive dysfunction (represented by the innermost circle), and these disease processes, in turn, may be modified by other factors (listed under the “policy” umbrella), which may be targets to maximize health outcomes and behaviors. Finally, public policy (including health policy and health care management) is the societal tool that can serve as the ultimate modifier of these various risk factors, and will determine the success of the major effort required to prevent these common co-morbid diseases that are increasingly likely to burden our aging population.

Now is a critical period for research in the field of metabolic and neurocognitive dysfunction. Major complementary efforts, including the Brain Research Through Advancing Innovative Neurotechnologies (BRAIN) initiative, the National Plan to Address Alzheimer’s Disease (NAPA), the Big Data to Knowledge (BD2K) initiative, and the Precision Medicine Initiative (PMI), will provide the financial support, tools, and scientific communities that are poised to substantially advance mechanistic research linking metabolic disease and neurocognitive dysfunction within the next dozen or so years. The scientific challenge here is to determine the mechanism(s) that explain the interactions between metabolism, the brain, and neurocognitive dysfunction, in order to identify methods for early detection of risk for metabolic and neurocognitive dysfunction and potential strategies and critical periods for prevention, mitigation, and intervention for these diseases. Figure 1 illustrates the most plausible biological mechanisms that may explain the bidirectional relationship between metabolic and neurocognitive dysfunction. These include mechanisms at the molecular, cellular, organ and tissue-based, and systemic levels, which may lead to metabolic and neurcognitive dysregulation that underlies disease processes contributing to obesity, insulin resistance, prediabetes, type 2 diabetes, and dementia (including VCID, VaD, AD, and others). In addition, these factors may be modified by other biological, behavioral, and environmental factors, which may be targets to optimize health outcomes. Public policy can serve as the ultimate modifier of these various risk factors, and will determine the success of the major effort required to prevent these common co-morbid diseases that are increasingly likely to burden our aging population.

An important step toward that goal is to take a highly heterogeneous group of individuals with metabolic and neurocognitive dysfunction and begin to cluster them into more homogeneous subgroups based on detailed clinical, metabolic, and neurocognitive data. This includes the need for more human brain specimens from individuals with good physiological characterization regarding diabetes, metabolic function, and neurocognitive function throughout the lifespan. This would allow preclinical researchers to test potential mechanisms based on distinct, relevant metabolic and neurocognitive abnormalities and develop prevention and treatment strategies targeting these phenotypes. Animal models will be an important contribution to these preclinical studies and should have comparable abnormalities in conserved domains compared to humans. For example, in rodents, it is reasonable to assume that learning and memory functions are somewhat comparable to humans, but it is unlikely that executive functions and decision-making (and the neural circuits that support these functions) are similar between species. Finally, a more homogeneous cluster of individuals with shared metabolic and neurocognitive resilience, risk or disease factors are allocated to targeted prevention and intervention efforts based on shared mechanisms to optimize prevention, mitigation, and treatment of the neurocognitive complications of diabetes and related metabolic dysfunction. In many ways, we already have the tools and technologies in place to perform deep phenotyping to guide future efforts for targeted, individualized therapies. Recently, it was discovered based on genetic analyses, that type 2 diabetes may in fact consists of 3 subtypes and that one specific subtype appears to be associated with neurological disease ( Li *et al.*, 2015). This type 2 diabetes subtype was also associated with cardiovascular disease, but not diabetic retinopathy or nephropathy, which implies that the mechanisms for neurocognitive complications of diabetes differ from complications in other end organs. There are related efforts, methods, and tools in development that could be leveraged for further discovery into the mechanisms at the interface of metabolic and neurocognitive function, including in the areas of neuroimaging ( Yeo *et al.*, 2016) and neuroimaging genetics ( Thompson *et al.*, 2014), web-based cognitive testing ( Germine *et al.*, 2012), and individualized neural and physiological phenotyping over time ( Poldrack *et al.*, 2015; Wang *et al.*, 2015; Yeo *et al.*, 2016).

## Supplementary material

**Supplementary File 1**

Workshop Program.

Details of participants and talks at the National Institute of Diabetes and Digestive and Kidney Diseases (NIDDK) The Intersection of Metabolic and Neurocognitive Dysfunction Workshop.

**Supplementary File 2**

Complete list of identified broad research needs identified at the workshop.
