# Supplementary material for: Complex mechanisms linking neurocognitive dysfunction to insulin resistance and other metabolic dysfunction
Source: F1000Res. 2016 Jun 2;5:353. Originally published 2016 Mar 15. [Version 2] doi: 10.12688/f1000research.8300.2 (PMC4897751; doi:10.12688/f1000research.8300.2)
Supplement: Supplementary file 1 [file f1000research-5-9550-s0000.tgz › 07adb16a-f676-4b71-b353-eef607c526e7.pdf]

Appendix. National Institute of Diabetes and Digestive and Kidney Diseases (NIDDK) The Intersection of Metabolic and Neurocognitive Dysfunction Workshop

National Institutes of Health (NIH) Campus, Building 31 Bethesda, MD
July 16 – 17, 2015

Agenda

Day One, July 16, 2015

12:30 – 1:00 p.m. **Welcome and Logistics**
Dr. Griffin P. Rodgers, Director, NIDDK
Dr. Luke Stoeckel

Plenary
Moderator: Dr. Dana Small

1:00 – 1:30 p.m. **Insulin and IGF-1 Signaling in the Brain and the Relationship to Diabetes and Neurodegenerative Disease**
Dr. C. Ronald Kahn

1:30 – 1:40 p.m. **Q and A**

Neurocognition and Metabolism in Obesity and Metabolic Disease
Moderator: Dr. Small

1:40 – 1:55 p.m. **Effect of Decrements in Circulating Glucose and Diabetes on Brain Function and Metabolism**
Dr. Robert Sherwin

1:55 – 2:00 p.m. **Q and A**

2:00 – 2:15 p.m. **Neural and Metabolic Regulation of Food Intake and Obesity: The Gut---Brain Axis as a Common Link between Metabolic and Cognitive Dysfunction**
Dr. Hans-Rudi Berthoud

2:15 – 2:20 p.m. **Q and A**

The Clinical Epidemiology of Diabetes and Neurocognition
Moderator: Dr. Samuel Gandy

2:20 – 2:35 p.m. **The Relationship between Diabetes, Aging, and Dementia**
Dr. Rachel Whitmer

2:35 – 2:40 p.m. **Q and A**

2:40 – 2:55 p.m. **Neuroimaging Correlates of Cognitive Dysfunction and Dementia in Diabetes**
Dr. Geert Jan Biessels

2:55 – 3:00 p.m. **Q and A**

3:00 – 3:15 p.m. **Diabetes, Brain Function, and Neuropathology**
Dr. Zoe Arvanitakis (Workshop Organizer)

3:15 – 3:20 p.m. **Q and A**

3:20 – 3:25p.m. **General Q and A**

3:25 – 3:45 p.m. **Break**

Basic Mechanisms of Metabolic and Neurocognitive Dysfunction
Moderator: Dr. Arvanitakis

3:45 – 4:00 p.m. **SORCS1: Genetic Defect in Protein Sorting Underlies Some Risk for Both Diabetes and Alzheimer's Disease**
Dr. Gandy (Workshop Organizer)

4:00 – 4:05 p.m. **Q and A**

4:05 – 4:20 p.m. **Insulin/IGF1 Signaling Mechanisms in Metabolism, Aging, and Neurodegenerative Disease**
Dr. Morris White

4:20 – 4:25 p.m. **Q and A**

4:25 – 4:40 p.m. **Blood-Brain Barrier Breakdown and Cognitive Decline**
Dr. Berislav Zlokovic

4:40 – 4:45 p.m. **Q and A**

Neurocognitive Mechanisms of Food Intake Behavior, Obesity, and Metabolic Dysfunction: I
Moderator: Dr. Alvaro Pascual-Leone

4:45 – 5:00 p.m. **The Mechanistic Links between "Western" Diet and Cognitive Dysfunction**
Dr. Scott Kanoski

5:00 – 5:05 p.m. **Q and A**

5:05 – 5:20 p.m. **Neurocognitive Adaptations Due to "Western" Diet and Obesity**
Dr. Dana Small (Workshop Organizer)

5:50 – 6:00 p.m. **Day 1 Summary and Take Home Points**
Dr. Kahn

6:00 p.m. **Adjournment**

Day Two, July 17, 2015

8:30 – 8:40 a.m. **Logistics (Conference Room 6)**
Dr. Stoeckel

Neurocognitive Mechanisms of Food Intake Behavior, Obesity, and Metabolic Dysfunction: II
Moderator: Dr. Small

8:40 – 8:55 a.m. **The Neural Basis of Self-Control: Rewards, Decisions, and Executive Functions in Obesity**
Dr. Lesley Fellows

8:55 – 9:00 a.m. **Q and A**

9:00 – 9:15 a.m. **Enhancing Neuroplasticity to Improve Brain Function in Diabetes**
Dr. Pascual-Leone (Workshop Organizer)

9:15 – 9:20 a.m. **Q and A**

Common Therapeutic Targets for Metabolic and Neurocognitive Dysfunction
Moderator: Dr. Zoe Arvanitakis

9:20 – 9:35 a.m. **Mechanisms of Neuronal Insulin Resistance in Alzheimer's Disease: New Targets for Repurposed Drugs**
Dr. Steve Arnold

9:35 – 9:40 a.m. **Q and A**

9:40 – 9:55 a.m. **Pharmacological and Nonpharmacological Interventions for Diabetes and Dementia: Effects on Cognition and CNS Biomarkers**
Dr. Suzanne Craft

9:55 – 10:00 a.m. **Q and A**

10:00 – 10:15 a.m. **Nonpharmacological Interventions for Obesity and Diabetes: Effects on Neurocognition**
Dr. John Gunstad

Novel Frontiers in the Study of the Brain and Metabolism
Moderator: Dr. Pascual-Leone

10:35 – 10:50 a.m. **Drosophila as a Model for Human Neurodegenerative Disease: Integrating the Microbiome**
Dr. Nancy Bonini

10:50 – 10:55 a.m. **Q and A**

10:55 – 11:10 a.m.	**Harnessing the Neuroimaging Arsenal for the Study of Neurocognition in the Context of Metabolic Disease**<br>*Dr. Peter Fox*
11:10 – 11:15 a.m.	**Q and A**
11:15 – 11:30 a.m.	**Assessment of Metabolic and Cognitive Interaction by Advanced Imaging Techniques over the Whole Life Span**<br>*Dr. Hubert Preissl*
11:30 – 11:35 a.m.	**Q and A**
11:35 – 11:50 a.m.	**Crowdsourcing to Study Cognition: New Insights into Human Cognitive Variation and How to Collect Large Samples for Understanding the Brain and Metabolic Disease**<br>*Dr. Laura Germine*
11:50 – 11:55 a.m.	**Q and A**
11:55 a.m. – 12:00 p.m.	**General Q and A**
12:00 – 12:30 p.m.	**Working Lunch**
12:30 – 2:00 p.m.	**Breakout Sessions**
	• **Topic 1 (Room 7)**
	• **Topic 2 (Room 8)**
	• **Topic 3 (Room 9)**
	• **Topic 4 (Room 6)**
	• **Topic 5 (Room 6)**

4

2:00 – 2:30 p.m. **General Discussion Following Breakout Sessions** (Room 6)

2:30 – 2:40 p.m. **Day Two Summary and Take Home Points**
Dr. Sherwin

2:40 – 3:40 p.m. **Overall Summary, Including Final Recommendations**
Moderators: Breakout Session Leaders (Drs. Gandy, Small, Kahn, Fox, Biessels, Arnold, and Arvanitakis)

3:40 – 3:50 p.m. **Concluding Remarks**
Dr. Sherwin

Day Three, July 18, 2015

8:30 a.m. – 1:00 p.m. Closed meeting: **Debrief and Plans for Future Efforts**
(Workshop Planning Committee Only)
