# Supplementary material for: Complex mechanisms linking neurocognitive dysfunction to insulin resistance and other metabolic dysfunction
Source: F1000Res. 2016 Jun 2;5:353. Originally published 2016 Mar 15. [Version 2] doi: 10.12688/f1000research.8300.2 (PMC4897751; doi:10.12688/f1000research.8300.2)
Supplement: Supplementary file 2 [file f1000research-5-9550-s0001.tgz › d7f447b7-4346-4c3c-8d42-4afde08004bf.docx]

Complete list of identified broad research needs identified at the workshop

- Improved tools and model systems for drug discovery and development
- Increased utilization of existing tools and model systems for mechanistic research
- Deep metabolic and neurocognitive phenotyping in human studies
- Improved biomarkers for use in human studies
- Increased utilization of existing biomarkers for human studies
- Improved human neuroimaging technologies
- Detailed metabolomics analysis of individual brain regions in both diabetes and dementia, using techniques such as imaging mass spectrometry
- Increased utilization of existing, novel human neuroimaging technologies
- Longitudinal human studies assessing cognition, metabolism and diet
- Human studies manipulating diet to determine causality and identify specific aspects of foods (e.g. macronutrients such as saturated fatty acids) that negatively impact brain and brain function
- An operational definition of “brain insulin resistance” and tools for measuring brain insulin signaling *in vivo*
- Better understanding of insulin action on astroglial versus neuronal cells as this relates to altered brain function in diabetes
- Development of a protocol to define and measure a “brain health index” for persons with metabolic dysfunction
- Need for harmonization and standardization across measures and protocols
- Combined human, animal, and cell culture studies
- Increased attention to gut-brain signaling, microbiome, and the relationship to obesity, metabolic disease, and neurocognitive dysfunction
- Evaluation of weight loss and diet for reversing or improving cognition
- Interdisciplinary teams collaborating on research at the intersection of metabolism and brain dysfunction
- Engaging and maintaining junior researchers in the field
