# Supplementary material for: Complex mechanisms linking neurocognitive dysfunction to insulin resistance and other metabolic dysfunction
Source: F1000Res. 2016 Jun 2;5:353. Originally published 2016 Mar 15. [Version 2] doi: 10.12688/f1000research.8300.2 (PMC4897751; doi:10.12688/f1000research.8300.2)
Supplement: Peer review history

# Peer review: Referee response for version 2

**DOI:** 10.5256/f1000research.9550.r14143

**Published:** 2016-06-03

**Authors:** Steven W Barger

**Affiliations:** 1 Department of Geriatrics, University of Arkansas for Medical Sciences, Little Rock, AR, USA; 2 Department of Neurobiology and Developmental Sciences, University of Arkansas for Medical Sciences, Little Rock, AR, USA; 3 Geriatric Research, Education & Clinical Center, Central Arkansas Veterans Healthcare System, Little Rock, AR, USA

The authors have embellished the manuscript with very well-considered - and considerate - responses to the reviewers' initial comments.

I have read this submission. I believe that I have an appropriate level of expertise to confirm that it is of an acceptable scientific standard.

# Peer review: Referee response for version 1

**DOI:** 10.5256/f1000research.8926.r13698

**Published:** 2016-05-06

**Authors:** Mahmoud Reza Azarpazhooh, Vladimir Hachinski

**Affiliations:** 1 Department of Clinical Neurological Sciences, University of Western Ontario, London, ON, Canada; 2 Department of Neurology, Mashhad University of Medical Sciences, Mashhad, Iran; 3 Department of Clinical Neurological Sciences (CNS), University of Western Ontario, London, ON, Canada

The authors offer a balanced review regarding the relationship between cognitive decline and insulin resistance. They cover a majority of important studies, providing excellent insights into our current knowledge, and more importantly gaps in definition, pathophysiology, and prevention and treatment of cognitive impairment related to diabetes.

One of the strongest parts of the paper is highlighting a probable reciprocal cause and effect relation between diabetes, leading to the cognitive decline, and neurodegenerative lesions in the brain, resulting in central insulin resistance. On the one hand, diabetes can contribute to dementia via a range of mechanisms, including vascular components ^1^, peripheral and central inflammatory response via activation of microglia ^2^, change in the blood brain barrier ^3^,  and neurostructural changes, both in white and gray matter ^4^. On the other hand, neurodegenerative changes, such as amyloid deposition in the brain, especially in the hypothalamus ^5^, may lead to insulin resistance and probably a central type of metabolic disorders and diabetes. This idea opens a new window in organized research for dementia and diabetes and at the same time underlines an urgent need to clarify the priorities in these vital fields.

This paper provides a long list of possible gaps and plans for further studies; however, some themes stand out:

1. **An urgent need for definitions, and collaborations:** Despite a relatively high incidence of diabetes and dementia and not surprisingly extensive research in these conditions, still no worldwide consensus exists on definitions, inclusion and exclusion criteria and clarity on some key words. This can be partially explained by focused research in diabetes and dementia, conducted in different disciplines in parallel and sometimes opposite directions. Considering diabetes as a systemic disease, affecting almost each and every part of human body, and cognitive decline, as a condition with a wide range of etiologies, and presentations, it is a high time to look at these common disorders in a more holistic, multi-dimensional vision and create data banks, including all available data and use similar definitions, and common vocabularies. Such national/international data banks may provide a better insight into epidemiology, pathophysiology, prevention,  and treatment of dementia and diabetes and at the same time can save our limited health resources.
2. **Experimental studies:** Animal studies play a significant role on our current knowledge in medicine. Despite their undeniable advantages in the evaluation of pathogenesis and treatment of several diseases, they still suffer from specific limitations, mainly due to high costs as well as the selection of the most appropriate animals. A similar significant gap can also be seen in animal studies of dementia and diabetes, using different animals, with limited sample sizes and lack of enough controls. Therefore, it may be worthwhile creating national/ international data banks for animal samples from both sexes with or without diseases, covering all possible aspects of animal studies, from behavioral and cognitive abilities to imaging and pathology. Such data banks could facilitate multidiscipline studies, save our resources and more importantly save on the number of sacrifized animals. This is an important step towards a better understanding of the pathogenesis of dementia and diabetes, and even starting new trials based on the underlying causes, such as anti-inflammatory medications.
3. **Human studies:** Using a common vocabulary in definition and categorization of diabetes and dementia, having national / international animal banks and finally a multidimensional approach toward such diseases with multiple and reciprocal presentations and etiologies may lead to optimal human studies and the creation of national/international human data banks. Undoubtedly, we need to provide more sensitive and specific instruments as well as tests to assess diabetes in the first stages and at the same time evaluate a range of cognitive abilities from executive functions, which is more common in vascular cognitive impairment, to memory abilities. In addition, such holistic vision can identify a range of comorbid and confounding disabilities in diabetes, from obesity and vascular diseases to important psychiatric conditions, such as depression. Finally, there is a need for designing multidiscipline studies to assess epidemiological aspects of dementia and diabetes, public health impact and practical clinical trials. Such studies can provide a great chance to detect diseases in pre-symptomatic phases and follow them longitudinally.   Multicenter trials with factorial design, using a combination of lifestyle modifications and medical intervention to diagnose as well as prevent/ treat diabetes and dementia would have paramount of importance in each and every community worldwide.

In summary, Stoeckel and colleagues highlight a significant gap of knowledge and consequently appropriate management, definitions, pathogenesis and treatment of dementia and diabetes. A combination of several comorbid and confounding factors and at the same time a possible bi-directional relationship between these common conditions emphasizes an urgent need for a more holistic multidimensional vision to tackle diabetes and dementia together.

We have read this submission. We believe that we have an appropriate level of expertise to confirm that it is of an acceptable scientific standard.

## References

[1] GorelickPBScuteriABlackSEDecarliCGreenbergSMIadecolaCLaunerLJLaurentSLopezOLNyenhuisDPetersenRCSchneiderJATzourioCArnettDKBennettDAChuiHCHigashidaRTLindquistRNilssonPMRomanGCSellkeFWSeshadriS: Vascular contributions to cognitive impairment and dementia: a statement for healthcare professionals from the american heart association/american stroke association. *Stroke*.2011;42(9) : 10.1161/STR.0b013e3182299496 2672-713 10.1161/STR.0b013e3182299496 21778438PMC3778669

[2] DeyAHaoSErionJRWosiski-KuhnMStranahanAM: Glucocorticoid sensitization of microglia in a genetic mouse model of obesity and diabetes. *J Neuroimmunol*.2014;269(1-2) : 10.1016/j.jneuroim.2014.01.013 20-7 10.1016/j.jneuroim.2014.01.013 24534266PMC3989932

[3] ShimizuFSanoYTominagaOMaedaTAbeMAKandaT: Advanced glycation end-products disrupt the blood-brain barrier by stimulating the release of transforming growth factor-β by pericytes and vascular endothelial growth factor and matrix metalloproteinase-2 by endothelial cells in vitro. *Neurobiol Aging*.2013;34(7) : 10.1016/j.neurobiolaging.2013.01.012 1902-12 10.1016/j.neurobiolaging.2013.01.012 23428182

[4] ReijmerYDBrundelMde BresserJKappelleLJLeemansABiesselsGJ: Microstructural white matter abnormalities and cognitive functioning in type 2 diabetes: a diffusion tensor imaging study. *Diabetes Care*.2013;36(1) : 10.2337/dc12-0493 137-44 10.2337/dc12-0493 22961577PMC3526236

[5] IshiiMIadecolaC: Metabolic and Non-Cognitive Manifestations of Alzheimer's Disease: The Hypothalamus as Both Culprit and Target of Pathology. *Cell Metab*.2015;22(5) : 10.1016/j.cmet.2015.08.016 761-76 10.1016/j.cmet.2015.08.016 26365177PMC4654127

# Response

**Published:** 2016-06-02

**Authors:** Luke Stoeckel

**Affiliations:** National Library of Medicine & NIH

Referee Reports: 6 May 2016 (MR Azarpazhooh and V Hachinski):

 We thank the referees for highlighting important strengths of our manuscript: “The authors offer a balanced review regarding the relationship between cognitive decline and insulin resistance. They cover a majority of important studies, providing excellent insights into our current knowledge, and more importantly gaps in definition, pathophysiology, and prevention and treatment of cognitive impairment related to diabetes.

 One of the strongest parts of the paper is highlighting a probable reciprocal cause and effect relation between diabetes, leading to the cognitive decline, and neurodegenerative lesions in the brain, resulting in central insulin resistance….”

Response: We agree with the sentiments expressed by these referees and have expanded the text to further emphasize the major themes identified at the workshop and identified by these referees, and we expanded discussion of these themes to better guide future research in this area (see pgs. 8-9). We request that the list of other research needs be moved to the supplementary materials section.

# Peer review: Referee response for version 1

**DOI:** 10.5256/f1000research.8926.r12962

**Published:** 2016-04-12

**Authors:** Steven W Barger

**Affiliations:** 1 Department of Geriatrics, University of Arkansas for Medical Sciences, Little Rock, AR, USA; 2 Department of Neurobiology and Developmental Sciences, University of Arkansas for Medical Sciences, Little Rock, AR, USA; 3 Geriatric Research, Education & Clinical Center, Central Arkansas Veterans Healthcare System, Little Rock, AR, USA

This rather comprehensive review of the connections between the metabolic syndrome associated with Type-2 diabetes mellitus (T2DM) and cognitive impairment was inspired by a workshop held by the National Institute of Diabetes and Digestive and Kidney Diseases (NIDDK).  The article is so thorough and well organized that one assumes the proceedings of that meeting must have been only the starting point and that the thoughts and opinions arising from that brainstorming session have been embellished with considerable diligence and reflection here.

Most of the points covered in this “white paper” are well reasoned and well researched.  And all the topics represent elements of pathology and pathogenesis that will be key to any faithful attempt to understand the intersection between T2DM, dementia, and milder forms of cognitive impairment.  Other authors have often succumbed to the temptation to focus on Alzheimer’s disease (AD) as the tag-word for these discussions, but one of the triumphs of this review is its more general consideration of dementia and sub-demented deficiencies as per their connections to energy substrates, both in the central nervous system and peripherally.  Indeed, the authors highlight a consideration that seems to be gaining in prominence and frequency in discussions of AD itself:  the fact that heterogeneity—especially within certain classifications of age, gender, and genetics—suggests not only multiple routes to AD, but also multiple disease endpoints.  Particularly within the “oldest-old” populations, APOE4-negative individuals, and high-risk populations such as military veterans, classic AD pathology correlates rather loosely with cognition.  To their credit, the authors discuss the possibility that metabolic derangement may yet be shown to be the driving force for at least some of these cases:

*"[I]t is still unclear if the relationship between diabetes and neurocognitive dysfunction and dementia is due to classic AD pathology, vascular processes such as infarcts or vessel pathology, other pathologies less closely related to AD or vascular disease (e.g., impaired brain insulin signaling), or some combination of these. Some have proposed that ‘all-cause’ dementia be employed in order to avoid the implication that we can classify etiologies accurately based on current knowledge."*

Another important point made here regards the direction of causality.  Hypotheses inspired by the earliest epidemiological links between dementia and T2DM presumed that complications of the latter drive the neuropathologies (mostly, those related to protein aggregation) that underlie AD and related dementias.  But a more accurate picture of the relevant events in AD progression has been afforded—largely through advances in neuroimaging tools—and this insight sets the stage for researchers to now say,

*"…the pathophysiological processes that lead to metabolic and neurocognitive dysfunction precede the development of clinical syndromes by years…. Indeed, AD neuropathology is detectable more than 10 years before the onset of the first clinical symptoms (Bateman et al., 2012) and pre-diabetes also precedes diabetes by years in most cases."*

In fact, data indicate that changes in amyloid β-peptide (Aβ) precede reductions in cerebral glucose utilization, the latter of which may be an important index of metabolic syndrome (below).  Supported by evidence from animal models, emerging trends in hypothesis-building include the supposition that CNS accumulation of Aβ, and perhaps other elements of neurodegeneration, are sufficient to perturb peripheral metabolism.  This allows consideration of the possibility that correlations between cognitive impairment and cortical amyloid burden are so capricious because an important modulatory (or even instigating) factor is the accumulation of Aβ in subcortical regions:

*"Therefore, it is plausible that AD pathology (amyloid-beta or Aβ, tauopathy) may be present in the hypothalamus prior to the onset of cognitive symptoms, and that this hypothalamic pathology could disrupt homeostatic functions such as energy balance and peripheral metabolism prior to the onset of detectable cognitive dysfunction (Ishii & Iadecola, 2015b)."*

It is also encouraging to see the inclusion of information on effects of obesity and diet that appear to be independent of glycemic regulation.  This is an intriguing body of data, considering the untoward effects of high-fat diets in rodents vis-à-vis the benefits that ketogenic diets and their mimics seem to have in humans ^1^.  It seems that metabolic impacts on brain health could benefit from better understanding of the effects that are brought to bear by shifts between the prominent energy-substrate molecules, perhaps through impacts on such substances that also impact neurotransmitter pools, such as glutamate and glutamine.  And it might be productive here to consider hypotheses regarding the finding that obesity in midlife is correlated with dementia manifest decades later ^2^, at a time when weight *loss* may be more common.

For all the salient points made in this white paper, the authors still find themselves perpetuating a conventional assumption that seems shortsighted in light of a substantial body of evidence; this is all the more disappointing because they consider what the implications would be “if metabolic disease impacts the brain in ways that differ from its effects on other end organs impacted by diabetes and other metabolic disease.”  This is almost certainly the case.  The traditional view that elevated glucose in the blood must, by necessity, produce elevated glucose in the brain ignores the existence of the blood-brain barrier (BBB); very little glucose can enter the cerebrum, or most other regions of the CNS, without regulated transport, the bulk of which is handled by glucose transporter 1 (GLUT1).  Thus, it was quite surprising to read a major conclusion in the paper that seems to have been based primarily on a single publication, and moreover, one devoted not to the brain but to the retina: 

*"Diabetes is associated with upregulation of the GLUT1 glucose transporter (Kumagai et al., 1996); glucose provides the original source of glutamate in the brain (Bradford & Thomas, 1969), and chronic hyperglycemia may thus lead to excessive glutamate and increased risk of excitotoxicity."*

This is subject to criticism on two counts:  1) Several studies show that AD and hyperglycemia are both associated with *reductions* in endothelial glucose transporters, likely reflecting—at least in the latter—a reactive feedback inhibition; this probably contributes to the diminished 2-deoxyglucose accumulation in both disorders.  2) Excitotoxicity is more likely to be a consequence of glucose deprivation; this is a well-established relationship ^3^^,^^4^, whereas the claim that high glucose elevates glutamate release is tenuous and unsupported by citations.  Publications documenting a diminution of GLUT1 levels in hyperglycemia and/or the converse, an elevation in hypoglycemia, comprise an overwhelming body of evidence ^5^^-^^10^.  The authors later provide some evidence suggesting effects on the BBB from metabolic abnormalities in rat models, particularly those brought on by diet, but data in humans is spotty and of unclear relevance to glucose delivery. 

Less serious or less consequential issues arose with regard to a few other elements in the paper:

*"Unlike other complications of diabetes…, neurocognitive complications of diabetes have not clearly been demonstrated to correlate with measures of peripheral glycemia (with the exception of very poor glycemic control, HbA1c > 10% and a potential role for glycemic variability) and there is only limited evidence for a modest association with other measures of peripheral glucose regulation…."*

Why would it be necessary—or indeed, even helpful—to exclude HbA1c levels and glycemic control from the calculus?  Which indices of peripheral glycemia would be more justifiable?  To be sure, 10% is a quite high level for HbA1c, but lower levels might show a detectable effect in an adequately powered study.  Moreover, HbA1c might not change dramatically under conditions of highly fluctuating blood-glucose concentrations, and yet such wild swings could easily produce intolerable strains on glucose-sensitive tissues—including brain endothelium—simply by virtue of their volatility.

The authors are to be applauded for their mention (however brief) of the apparent paradox between the ill effects of pathological insulin resistance versus the benefits of experimentally interrupting insulin signaling, at least in *C. elegans*.  It should be noted that similar benefits (e.g., on live span) accrue in mammals from inhibition of signaling for insulin-like growth factor (IGF), and *C. elegans* have only one peptide to accomplish the roles of both insulin and IGF.  Nevertheless, the conundrum is one that has vexed experimental gerontologists for decades, owing in part to the similar benefits afforded by attention of the insulin/IGF signaling (IIS) pathway and by dietary restriction, the latter of which certainly reduces overall insulin production.  One should not expect the authors to have cracked this tough nut, but important rumination might result from a slightly more complete discussion of the differences between reactive (feedback-mediated) insulin resistance and the proactive sort effected by empirical manipulation of IIS. 

Also given short shrift here is the undeniable connection of inflammation to both metabolic syndrome and neurocognitive disorders.  This is obviously an underappreciated link that deserves greater attention among scientists seeking to synthesize these fields; reviewers of grants and papers continue to express befuddlement when the topic of neuroinflammation is broached in this context.  Given the overwhelming evidence for inflammation-related events in the etiologies of both T2DM and AD, it is almost certain to contribute to their coincidence.  In fact, compelling evidence has supported just this sort of causal involvement in animal models ^11^.  The authors have included the word “inflammation,” distributed diffusely in the paper; perhaps it would create the appropriate emphasis if some of these references were consolidated in one or two paragraphs along with more specific information, e.g., the seminal role of IKK2 in insulin resistance ^12^ and Aβ effects ^13^. 

Finally, the paper’s conclusion with “Some broad research needs” is doubtlessly faithful to the output of the workshop’s deliberations, but many of the points outlined in this list come across as rather vague.  Without further elaboration, it is not clear what gaps in knowledge some of them would address.  One wonders if several could be combined and embellished with a bit more elaboration. 

In its overall contribution, this white paper provides a very useful and much-needed synthesis of data and concepts regarding the connections between cognition and metabolic derangement.  Modifiable elements of lifestyle and environment have recently emerged as major considerations in the prevention of dementia.  Stoeckel *et al. *have enhanced the conversation by broadening it to other categories of cognitive impairment.  In addition, they largely succeeded in summarizing the current state of fields that can seem disparate, and they highlight opportunities to draw these fields closer.

I have read this submission. I believe that I have an appropriate level of expertise to confirm that it is of an acceptable scientific standard, however I have significant reservations, as outlined above.

## References

[1] RegerMAHendersonSTHaleCCholertonBBakerLDWatsonGSHydeKChapmanDCraftS: Effects of beta-hydroxybutyrate on cognition in memory-impaired adults. *Neurobiol Aging*.2004;25(3) : 10.1016/S0197-4580(03)00087-3 311-4 10.1016/S0197-4580(03)00087-3 15123336

[2] AnsteyKJCherbuinNBudgeMYoungJ: Body mass index in midlife and late-life as a risk factor for dementia: a meta-analysis of prospective studies. *Obes Rev*.2011;12(5) : 10.1111/j.1467-789X.2010.00825.x e426-37 10.1111/j.1467-789X.2010.00825.x 21348917

[3] MonyerHGoldbergMPChoiDW: Glucose deprivation neuronal injury in cortical culture. *Brain Res*.1989;483(2) :347-54 256515010.1016/0006-8993(89)90179-0

[4] SinghSPEhmannSSnyderAK: Ethanol and glucose-deprivation neurotoxicity in cortical cell cultures. *Metabolism*.1994;43(9) :1108-13 791611710.1016/0026-0495(94)90052-3

[5] GjeddeACroneC: Blood-brain glucose transfer: repression in chronic hyperglycemia. *Science*.1981;214(4519) :456-7 702743910.1126/science.7027439

[6] McCallALMillingtonWRWurtmanRJ: Metabolic fuel and amino acid transport into the brain in experimental diabetes mellitus. *Proc Natl Acad Sci U S A*.1982;79(17) :5406-10 675294710.1073/pnas.79.17.5406PMC346906

[7] MatthaeiSHorukROlefskyJM: Blood-brain glucose transfer in diabetes mellitus. Decreased number of glucose transporters at blood-brain barrier. *Diabetes*.1986;35(10) :1181-4 353084710.2337/diab.35.10.1181

[8] PardridgeWMTrigueroDFarrellCR: Downregulation of blood-brain barrier glucose transporter in experimental diabetes. *Diabetes*.1990;39(9) :1040-4 238418710.2337/diab.39.9.1040

[9] LutzAJPardridgeWM: Insulin therapy normalizes GLUT1 glucose transporter mRNA but not immunoreactive transporter protein in streptozocin-diabetic rats. *Metabolism*.1993;42(8) :939-44 834581610.1016/0026-0495(93)90004-8

[10] CornfordEMHymanSCornfordMEClare-SalzlerM: Down-regulation of blood-brain glucose transport in the hyperglycemic nonobese diabetic mouse. *Neurochem Res*.1995;20(7) :869-73 747768110.1007/BF00969700

[11] ClarkeJRLyra E SilvaNMFigueiredoCPFrozzaRLLedoJHBeckmanDKatashimaCKRazolliDCarvalhoBMFrazãoRSilveiraMARibeiroFCBomfimTRNevesFSKleinWLMedeirosRLaFerlaFMCarvalheiraJBSaadMJMunozDPVellosoLAFerreiraSTDe FeliceFG: Alzheimer-associated Aβ oligomers impact the central nervous system to induce peripheral metabolic deregulation. *EMBO Mol Med*.2015;7(2) : 10.15252/emmm.201404183 190-210 10.15252/emmm.201404183 25617315PMC4328648

[12] TantiJFJagerJ: Cellular mechanisms of insulin resistance: role of stress-regulated serine kinases and insulin receptor substrates (IRS) serine phosphorylation. *Curr Opin Pharmacol*.2009;9(6) : 10.1016/j.coph.2009.07.004 753-62 10.1016/j.coph.2009.07.004 19683471

[13] LiuYLiuXHaoWDeckerYSchomburgRFülöpLPasparakisMMengerMDFassbenderK: IKKβ deficiency in myeloid cells ameliorates Alzheimer's disease-related symptoms and pathology. *J Neurosci*.2014;34(39) : 10.1523/JNEUROSCI.1348-14.2014 12982-99 10.1523/JNEUROSCI.1348-14.2014 25253847PMC6608331

# Response

**Published:** 2016-06-02

**Authors:** Luke Stoeckel

**Affiliations:** National Library of Medicine & NIH

Referee Reports: 12 April 2016 (SW Barger):

 We appreciate that Dr. Barger felt this review was “so thorough and well organized that one assumes the proceedings of that meeting must have been only the starting point and that the thoughts and opinions arising from that brainstorming session have been embellished with considerable diligence and reflection here.”  We also thank the referee for concluding that “In its overall contribution, this white paper provides a very useful and much-needed synthesis of data and concepts regarding the connections between cognition and metabolic derangement.  Modifiable elements of lifestyle and environment have recently emerged as major considerations in the prevention of dementia.  Stoeckel *et al. *have enhanced the conversation by broadening it to other categories of cognitive impairment.  In addition, they largely succeeded in summarizing the current state of fields that can seem disparate, and they highlight opportunities to draw these fields close”.

Point 1: “It is also encouraging to see the inclusion of information on effects of obesity and diet that appear to be independent of glycemic regulation.  This is an intriguing body of data, considering the untoward effects of high-fat diets in rodents vis-à-vis the benefits that ketogenic diets and their mimics seem to have in humans ^1^.  It seems that metabolic impacts on brain health could benefit from better understanding of the effects that are brought to bear by shifts between the prominent energy-substrate molecules, perhaps through impacts on such substances that also impact neurotransmitter pools, such as glutamate and glutamine.  And it might be productive here to consider hypotheses regarding the finding that obesity in midlife is correlated with dementia manifest decades later ^2^, at a time when weight *loss* may be more common.”

Response: Text modified to make this important point clear (see p. 6, paragraph 1). “Compared to adults with normal body mass index (BMI = 18.5 – 24.9), overweight and obese adults in midlife (ages 40-59 years) have a relative risk (RR) of 1.35 (overweight) and 2.04 (obese) for AD and 1.26 (overweight) and 1.64 (obese) of any cause dementia (Anstey et al. 2011). This association between overweight and obesity is not observed in late life (ages 60 years or older) at a time when weight loss is more common and a risk factor for dementia (Anstey et al. 2011).”

Point 2: “For all the salient points made in this white paper, the authors still find themselves perpetuating a conventional assumption that seems shortsighted in light of a substantial body of evidence; this is all the more disappointing because they consider what the implications would be “if metabolic disease impacts the brain in ways that differ from its effects on other end organs impacted by diabetes and other metabolic disease.”  This is almost certainly the case.  The traditional view that elevated glucose in the blood must, by necessity, produce elevated glucose in the brain ignores the existence of the blood-brain barrier (BBB); very little glucose can enter the cerebrum, or most other regions of the CNS, without regulated transport, the bulk of which is handled by glucose transporter 1 (GLUT1).  Thus, it was quite surprising to read a major conclusion in the paper that seems to have been based primarily on a single publication, and moreover, one devoted not to the brain but to the retina: 

*"Diabetes is associated with upregulation of the GLUT1 glucose transporter (Kumagai et al., 1996); glucose provides the original source of glutamate in the brain (Bradford & Thomas, 1969), and chronic hyperglycemia may thus lead to excessive glutamate and increased risk of excitotoxicity."*

 This is subject to criticism on two counts:  1) Several studies show that AD and hyperglycemia are both associated with *reductions* in endothelial glucose transporters, likely reflecting—at least in the latter—a reactive feedback inhibition; this probably contributes to the diminished 2-deoxyglucose accumulation in both disorders.  2) Excitotoxicity is more likely to be a consequence of glucose deprivation; this is a well-established relationship ^3^ ^,^ ^4^, whereas the claim that high glucose elevates glutamate release is tenuous and unsupported by citations.  Publications documenting a diminution of GLUT1 levels in hyperglycemia and/or the converse, an elevation in hypoglycemia, comprise an overwhelming body of evidence ^5^ ^-^ ^10^.  The authors later provide some evidence suggesting effects on the BBB from metabolic abnormalities in rat models, particularly those brought on by diet, but data in humans is spotty and of unclear relevance to glucose delivery.”

Response: We agree with the reviewer and have reorganized the section on “Mechanisms linking diabetes to dementia (p. 3)” and have modified discussion of the relationship between peripheral hyperglycemia and CNS hyperglycemia, GLUT1 glucose transporter, and excitotoxicity in response to the reviewer’s feedback. Specifically, we have indicated that excitotoxicity in the brain may be more closely related to glucose deprivation via reductions in endothelial glucose transporters.

 The following text can be found on p. 4, paragraph 3: “Chronic hyperglycemia has been associated with reductions in endothelial glucose transporters such as GLUT1 that could lead to glucose deprivation and excitotoxicity, and could account for some of the neurocognitive complications of diabetes (Gjedde and Crone 1981; Matthaei et al. 1986). This is unlike diabetic retinopathy, which has been associated with upregulation of GLUT1 glucose transporter ( Kumagai *et al.*, 1996) and is another example of how diabetic complications in the brain may differ from other organ systems.”

Point 3: *"Unlike other complications of diabetes…, neurocognitive complications of diabetes have not clearly been demonstrated to correlate with measures of peripheral glycemia (with the exception of very poor glycemic control, HbA1c > 10% and a potential role for glycemic variability) and there is only limited evidence for a modest association with other measures of peripheral glucose regulation…."*

 “Why would it be necessary—or indeed, even helpful—to exclude HbA1c levels and glycemic control from the calculus?  Which indices of peripheral glycemia would be more justifiable?  To be sure, 10% is a quite high level for HbA1c, but lower levels might show a detectable effect in an adequately powered study.  Moreover, HbA1c might not change dramatically under conditions of highly fluctuating blood-glucose concentrations, and yet such wild swings could easily produce intolerable strains on glucose-sensitive tissues—including brain endothelium—simply by virtue of their volatility.”

Response: We appreciate the need for clarification on this comment. We did not intend to suggest that HbA1c or glycemic variability were unimportant, and we agree that some existing studies may be underpowered to detect effects at lower levels of hyperglycemia (i.e., HbA1c < 10%). We have modified our text to emphasize future studies should not ignore the roles of HbA1c and glycemic variability, and there is a need for more adequately powered studies to clarify the roles for these variables in cognitive impairment and dementia. The following text can be found on p. 5, paragraph 2: “It is possible that lower levels of hyperglycemia (i.e., HbA1c < 10%) may have detectable effects on neurocognitive function in adequately powered studies or that HbA1c levels may not reflect highly fluctuating blood glucose concentrations, which could have independent deleterious effects on glucose-sensitive tissues such as brain endothelium.”

Point 4: “The authors are to be applauded for their mention (however brief) of the apparent paradox between the ill effects of pathological insulin resistance versus the benefits of experimentally interrupting insulin signaling, at least in *C. elegans*.  It should be noted that similar benefits (e.g., on live span) accrue in mammals from inhibition of signaling for insulin-like growth factor (IGF), and *C. elegans* have only one peptide to accomplish the roles of both insulin and IGF.  Nevertheless, the conundrum is one that has vexed experimental gerontologists for decades, owing in part to the similar benefits afforded by attention of the insulin/IGF signaling (IIS) pathway and by dietary restriction, the latter of which certainly reduces overall insulin production.  One should not expect the authors to have cracked this tough nut, but important rumination might result from a slightly more complete discussion of the differences between reactive (feedback-mediated) insulin resistance and the proactive sort effected by empirical manipulation of IIS.” 

Response: We appreciate this important point from the reviewer; however, this did not need receive substantial discussion at the workshop and, given the complexity of this issue, would require substantial more attention beyond the scope of the current paper. We have acknowledged this point in the manuscript. The following text can be found on p.5, paragraph 2: “The apparent paradox between pathological insulin resistance and the benefits of acute disruption of insulin signaling is a complex topic requiring further research attention.”

Point 5: “Also given short shrift here is the undeniable connection of inflammation to both metabolic syndrome and neurocognitive disorders.  This is obviously an underappreciated link that deserves greater attention among scientists seeking to synthesize these fields; reviewers of grants and papers continue to express befuddlement when the topic of neuroinflammation is broached in this context.  Given the overwhelming evidence for inflammation-related events in the etiologies of both T2DM and AD, it is almost certain to contribute to their coincidence.  In fact, compelling evidence has supported just this sort of causal involvement in animal models ^11^.  The authors have included the word “inflammation,” distributed diffusely in the paper; perhaps it would create the appropriate emphasis if some of these references were consolidated in one or two paragraphs along with more specific information, e.g., the seminal role of IKK2 in insulin resistance ^12^ and Aβ effects ^13^.”

Response: We agree with the reviewer that this is another important area for further research attention. However, inflammation in the context of diabetes and dementia was not the primary focus of the workshop; therefore, we refer the reader to recent reviews for more in depth attention to this important topic. The following text can be found on p.5, paragraph 1: “Inflammation-related events in the periphery and brain may be another important factor explaining the connection between diabetes and neurocognitive dysfunction (see (Ferreira et al. 2014; De Felice and Lourenco 2015) for recent reviews of this topic).”

Point 6: “Finally, the paper’s conclusion with “Some broad research needs” is doubtlessly faithful to the output of the workshop’s deliberations, but many of the points outlined in this list come across as rather vague.  Without further elaboration, it is not clear what gaps in knowledge some of them would address.  One wonders if several could be combined and embellished with a bit more elaboration.”

Response: We apologize for being overly vague in this section and have revised this section to provide more clarity.
